# Psychometric Properties of the Chinese Version of the Brief-Mindful Self-Care Scale: A Translation and Validation Study

**DOI:** 10.3389/fpsyg.2021.715507

**Published:** 2021-08-13

**Authors:** Zhen Yang, Fengmin Chen, Siqi Liu, Ming Dai, Huijun Zhang

**Affiliations:** ^1^Department of Nursing, Jinzhou Medical University, Jinzhou, China; ^2^Department of Nursing, The First Affiliated Hospital, Jinzhou Medical University, Jinzhou, China; ^3^Department of Nursing, Changchun University of Chinese Medicine, Changchun, China; ^4^Department of Nursing, Jiamusi University, Jiamusi, China

**Keywords:** mindfulness, self-care, compassion fatigue, hospice nurses, factor analysis

## Abstract

**Objective:** This study aimed to translate the Brief-Mindful Self-Care Scale (B-MSCS) into Chinese and validate its reliability and validity among hospice nurses.

**Methods:** A total of 510 hospice nurses were recruited from three provinces in China. The reliability of the translated scale was measured by internal consistency, split-half reliability, and test-retest reliability. The validity of the translated scale was evaluated by expert consultation, exploratory factor analysis, and confirmatory factor analysis.

**Results:** The Cronbach's α value of the Chinese version of B-MSCS was 0.920, and the Cronbach's α value of the dimensions ranged from 0.850 to 0.933. The split-half reliability and test-retest reliability were 0.770 and 0.723, respectively. Furthermore, the content validity index of the scale (S-CVI) was 0.946. The 6-factor structure, supported by the eigenvalues, total variance explained, and scree plot were obtained by using exploratory factor analysis. Moreover, as a result of the confirmatory factor analysis, the model fitting indexes were all in the acceptable range.

**Conclusion:** The Chinese version of B-MSCS had suitable reliability and validity among hospice nurses. The developed scale will evaluate the level of mindful self-care of Chinese hospice nurses, providing an opportunity for development of targeted educational plans. Each item is a direct guide for hospice nurses to develop their mindful self-care practice.

## Introduction

Hospice nurses often have compassion satisfaction in their hospice nursing practice. However, long-term exposure to death situations can cause compassion fatigue (Hotchkiss and Cook-Cottone, 2019), often described as the negative cost of hospice care (Barrett et al., 2019). The negative emotions can cause them to experience low energy levels, difficulty concentrating, unwanted images or thoughts, insomnia, stress, and desensitization, and even potentially substance abuse, depression and suicide in the long term (Pérez-García et al., 2021). The study results showed that 18% of hospice nurses met the criteria for compassionate fatigue and suffered from different degrees of insomnia and depression (Kase et al., 2019). Therefore, it is urgent for hospice nurses to find correlative factors to counter the negative emotions caused by compassion fatigue in the nursing practice.

Mindfulness, as a form of self-care, is defined as paying attention to what is happening in the mind, body and external environment with an attitude of curiosity and kindness (Tomlinson et al., 2018; Hotchkiss and Cook-Cottone, 2019), which aims to reduce compassion fatigue and promote compassion satisfaction (Hotchkiss, 2018). Mindfulness can be formal (meditation, yoga, tai chi, and other activities) or informal (walking, washing dishes, interacting with others) (Cook-Cottone, 2015). The related studies have found that mindfulness practitioners showed more stress management and coping techniques than a relaxed or self-affirming control group (Lucas-Thompson et al., 2020). Moreover, mindfulness can reduce stress by improving emotional regulation, leading to better mood and better ability to deal with stress (Wheeler et al., 2018; Li and Bressington, 2019). Also, mindfulness has always been regarded as an effective adjuvant treatment for depression. Compared with guided imagery relaxation, brief mindfulness training helped participants with depression regulate their emotions and better manage the negative effects of depression (Costa and Barnhofer, 2016). Crucially, mindfulness even worked for people dealing with the most critical depressive symptom of all: suicidal thoughts (Anastasiades et al., 2017; Raj et al., 2019). In addition to many mental health benefits, mindfulness can also improve physical health. Mindfulness practices can enhance or increase various health-related behaviors, such as having regular checkups, being physically active, using seat belts in cars, and avoiding smoking and alcohol (Karyadi and Cyders, 2015; Cook-Cottone, 2016; Fanning et al., 2018; Koppel et al., 2018). In addition, mindfulness is associated with improved cardiovascular health, as it is associated with lower smoking rates, more exercise and a better BMI (Barnett and Ruiz, 2018; López-Alarcón et al., 2020; Weng et al., 2021).

Before the impact of mindfulness on self-care, the traditional self-care model was also significant for hospice nurses (Matarese et al., 2018). However, it provided limited present-moment integration and saw self-care as a task rather than a healthy lifestyle (Hotchkiss, 2018; Cuartero and Campos-Vidal, 2019). Therefore, from the salient features of traditional self-care and arising out of the theory of attunement and embodied self-regulation (Cook-Cottone, 2015; Piran, 2015), the Mindful Self-Care Scale (MSCS), which integrated mindfulness with traditional self-care was developed (Cook-Cottone and Guyker, 2018). In 2017, the scale was developed into a brief version (B-MSCS) and validated among hospice nurses and health care professionals (Hotchkiss and Cook-Cottone, 2019). Mindful self-care is a repeated process of assessing mindfulness consciousness and the specific practice of self-care to meet the individual's internal and external needs in work and life (Hotchkiss and Cook-Cottone, 2019). The development of the MSCS and B-MSCS has arisen out of this need to assess participants' level of mindful self-care in their daily practice (Cook-Cottone and Guyker, 2018; Hotchkiss and Cook-Cottone, 2019). Despite the highlighted benefits of mindful self-care in the physiology and psychology of the individual, but research on mindful self-care is still incomplete in China, especially lacking a tool to evaluate the level of mindful self-care among hospice nurses. In this study, the English version of B-MSCS was introduced into China through translation and cultural adjustments. Moreover, we put forward a research question whether and to what extent the Chinese version of the B-MSCS exhibit satisfactory psychometric properties among hospice nurses?

## Methods

### Participants

This multicenter cross-sectional study was conducted and involved three provinces—Liaoning, Jilin and Heilongjiang, China—from February 2021 to April 2021. The sample size was determined using the general rule for factor analytic procedure that requires a minimum of three respondents per item (Kline, 1998), but a larger sample is desirable. In this study, 10 respondents per item were required to ensure the accuracy of exploratory factor analysis and confirmatory factor analysis. Therefore, participants were recruited by convenience sampling from hospitals with the assistance of nursing directors and consisted of 510 hospice nurses. Inclusion criteria required that participants were registered nurses engaged in hospice care and volunteered to participate in this study.

### Procedure

#### Data Collection Procedure

The researchers were divided into three groups (five people per group). After receiving relevant training, researchers went to three provinces respectively and recruited participates with the assistance of nursing directors. Participants completed the translated scale anonymously in a quiet classroom arranged by head nurses. To evaluate test-retest reliability, 40 hospice nurses were asked to complete the translated scale again after 2 weeks.

#### Scale Translation Procedure

**Figure 1:**
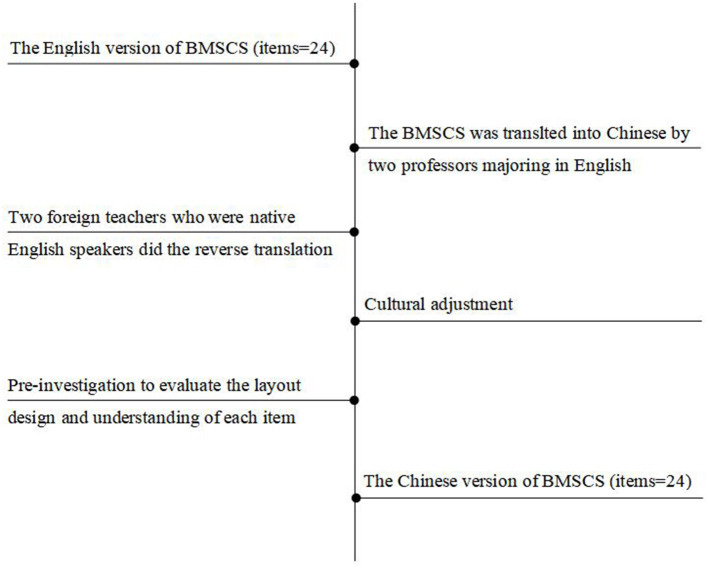
The translation procedure for Chinese version of the B-MSCS.

Our translation work has obtained professor Cook-Cottone's permission. First, B-MSCS was translated into Chinese by two Chinese professors majoring in English. Then, two foreign teachers who were native English speakers did the reverse translation. In addition, psychological experts were invited to cultural adjustments for the translated scale to make the items more compatible with Chinese expressions habits. Finally, 10 hospice nurses were selected to conduct a preliminary survey using convenience sampling, and were invited to evaluate the layout design and understanding of each item. Hospice nurses all indicated that the scale structure was clear and the items were easy to understand. The translation procedure is shown in Figure 1.

### Measures

#### Background Characteristics

A thorough literature review was conducted, after which the team designed the General Demographic Characteristics Questionnaire. Participants were required to complete six items by self-reporting: age, gender, education level, marital status, site, and professional experience.

#### Brief-Mindful Self-Care (BMSC) Scale

The levels of mindful self-care were measured by the Brief-Mindful Self-Care Scale (B-MSCS) developed by Hotchkiss and Cook-Cottone (Cook-Cottone and Guyker, 2018). The scale includes 24 items measured on a Likert scale from one to five, corresponding to (one) never, (two) rarely, (three) sometime, (four) often, and (five) regularly. Six domains were evaluated: mindful relaxation, physical care, self-compassion and purpose, supportive relationships, supportive structure, and mindful awareness. The score ranged from 24 to 120. The higher the total score, the higher the level of mindful self-care. The Cronbach's α value of the dimensions were 0.77–0.86.

### Data Analyses

#### Items Analysis

The total score was ranked from high to low and the relationship between the first 27% (high-score group) and the last 27% (low-score group) was analyzed to judge whether the translated scale has suitable discrimination. The correlation between the items and the translated scale and the Cronbach's α coefficient if item deleted are analyzed to evaluate whether each item of the translated scale can be retained.

#### Reliability Analysis

Cronbach's α value of the translated scale and its dimensions was calculated to assess the internal consistency reliability. According to the order of oddness and evenness, the items of the translated scale were divided into two parts, and the correlation between the results on both sides was calculated to evaluate the split-half reliability. Two weeks later, the translated scale was used to assess the test-retest reliability of the scale among 40 hospice nurses.

#### Validity Analysis

**Figure 2:**
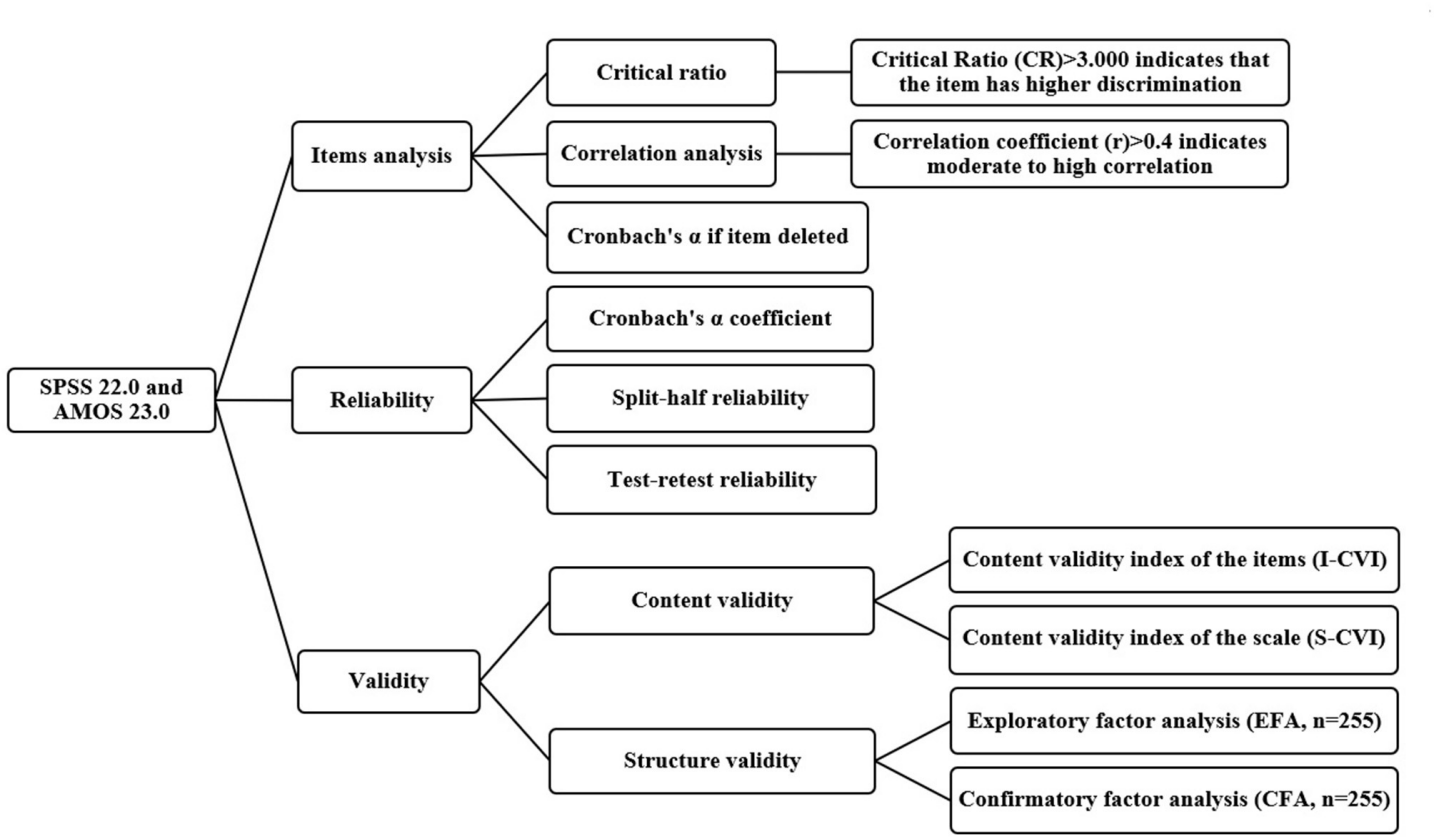
The data analysis procedure for Chinese version of the B-MSCS.

Seven experts were invited to evaluate the content validity of the scale using the Delphi method. The content validity index (I-CVI) of the items and the content validity index (S-CVI) of the translated scale were calculated. The exploratory factor analysis (EFA) and confirmatory factor analysis (CFA) were performed to evaluate the underlying factor structure of the translated scale. The sample of 510 cases was randomly divided into two groups, one (*n* = 255) for EFA and the other (*n* = 255) for CFA. Both groups' characteristics are substantially similar. Kaiser-Meyer-Olkin (KMO) and Bartlett test of sphericity were used to judge the rationality of using principal component analysis (PCA) with varimax rotation in EFA. Only when the Bartlett test of sphericity was significant (*P* < 0.05) and the KMO was >0.60, the dataset was considered appropriate for PCA. Analysis of Moment Structure (AMOS) was used in CFA and analyzing whether the fitting index of the model is suitable. The data analysis procedure is shown in Figure 2.

### Ethical Approval

Prior to the investigation, participants were informed of the purpose and significance of this study and signed an informed consent. Moreover, all returned questionnaires were anonymous. All procedures were performed with the 1964 Helsinki declaration, and the study protocol was approved by the Ethics Committee of the Jinzhou Medical University.

## Results

### Descriptive Statistics

This study included 510 hospice nurses: 103 males (20.2%) and 407 females (79.8%). Participants aged 25–34 years accounted for 58.4%. More than half (64.1%) of the participants were married; 44.9% of the participants had a junior college education. The proportion of participants who came from Liaoning province was the largest (41.4%); for the years of professional experience, 41.6% of participants have been in hospice care for 6–10 years. Other sociodemographic information are shown in Table 1.

**Table 1:** Frequency distribution of demographic characteristics (*n* = 510).

**Factors**	**Group**	***n***	**%**
Age	18–24	62	12.2
	25–34	298	58.4
	35–44	134	26.3
	≥45	16	3.1
Sex	Male	103	20.2
	Female	407	79.8
Education level	Technical secondary school education	69	13.5
	Junior college education	229	44.9
	Undergraduate education	181	35.5
	Postgraduate education	31	6.1
Marital status	Unmarried	171	33.5
	Married	313	61.4
	Divorced/Widowed	26	5.1
Site	Liaoning province	211	41.4
	Jilin Province	141	27.6
	Heilongjiang province	158	31.0
Professional experience (year)	1–5	163	32.0
	6–10	212	41.6
	11–15	69	13.5
	16–20	43	8.4
	≥20	23	4.5

### Item Analysis

The critical ratio (CR) >3.000 indicated the higher discriminability of items. The CR of 24 items in the translated scale was 7.780–19.567, which indicated that the discrimination of each item was good. The scores of each item were positively correlated with the total score (*r* = 0.404–0.740, *P* < 0.001), indicated that each item was moderately correlated with the scale. After deleting each item, Cronbach's α value of the translated scale was 0.914–0.919, which does not exceed Cronbach's α value of the scale (0.920) (Table 2).

**Table 2:** Item analysis for Chinese version of the B-MSCS.

**Item**	**Item score**	**Critical ratio**	**Correlation coefficient between item and total score**	**Cronbach's Alpha if item deleted**
MR-1	2.02 ± 0.99	19.567	0.737	0.914
MR-2	1.92 ± 0.91	12.130	0.651	0.917
MR-3	2.02 ± 1.04	18.453	0.721	0.914
MR-4	2.01 ± 1.04	18.559	0.740	0.914
PC-1	2.38 ± 0.92	15.306	0.616	0.917
PC-2	2.83 ± 0.82	9.244	0.459	0.919
PC-3	2.83 ± 0.86	19.260	0.691	0.915
PC-4	2.39 ± 0.91	17.494	0.657	0.916
PC-5	2.85 ± 0.80	12.753	0.559	0.918
SCP-1	3.00 ± 0.90	17.067	0.676	0.915
SCP-2	3.34 ± 0.86	13.734	0.682	0.917
SCP-3	2.97 ± 0.93	16.923	0.675	0.915
SCP-4	2.64 ± 0.89	14.865	0.601	0.917
SR-1	2.90 ± 0.84	18.454	0.646	0.916
SR-2	2.92 ± 0.78	18.716	0.651	0.916
SR-3	2.89 ± 0.83	19.378	0.686	0.915
SR-4	2.93 ± 0.78	19.456	0.674	0.915
SS-1	2.59 ± 0.80	10.936	0.444	0.918
SS-2	3.47 ± 0.66	7.780	0.430	0.919
SS-3	2.96 ± 0.74	12.145	0.481	0.919
SS-4	2.88 ± 0.74	11.744	0.504	0.918
MA-1	3.42 ± 0.80	11.507	0.521	0.918
MA-2	3.30 ± 0.85	8.843	0.404	0.917
MA-3	3.39 ± 0.78	10.264	0.456	0.919

*MR, Mindful Relaxation; PC, Physical Care; SCP, Self-Compassion and Purpose; SR, Supportive Relationships; SS, Supportive Structure; MA, Mindful Awareness*.

### Reliability Analysis

The Cronbach's α value of the translated scale was 0.920, and the Cronbach's α value of the dimensions ranged from 0.850 to 0.933. In addition, the split-half reliability was 0.770 and after 2 weeks, 40 hospice nurses were randomly selected for retesting, and the test-retest reliability was 0.732 (Table 3).

**Table 3:** Reliability analysis for Chinese version of the B-MSCS.

**The scale and** **Its dimension**	**Score**	**Cronbach's Alpha**	**Split-half** **reliability**	**Test-retest** **reliability**
**The B-MSCS**	66.85 ± 12.20	0.920	0.770	0.732
Mindful relaxation	7.98 ± 3.58	0.919		
Physical care	13.27 ± 3.63	0.899		
Self-Compassion and Purpose	11.95 ± 3.03	0.869		
Supportive relationships	11.64 ± 2.95	0.933		
Supportive structure	11.89 ± 2.05	0.850		
Mindful awareness	10.11 ± 2.14	0.853		

### Validity Analysis

#### Content Validity Analysis

Seven experts were invited to evaluate the content validity of the translated scale. The results showed that the I-CVI of the translated scale was 0.857–1.000 (Table 4), and the S-CVI was 0.946.

**Table 4:** Content validity analysis for Chinese version of the B-MSCS.

**Item**	**Experts (score)**							**I-CVI**
	**1**	**2**	**3**	**4**	**5**	**6**	**7**	
MR-1	1	1	0	1	1	1	1	0.857
MR-2	1	1	1	1	1	1	1	1.000
MR-3	1	1	1	1	1	1	1	1.000
MR-4	1	1	1	1	1	1	1	1.000
PC-1	1	0	1	1	1	1	1	0.857
PC-2	1	1	1	1	1	1	1	1.000
PC-3	1	1	1	1	1	1	1	1.000
PC-4	1	1	0	1	1	1	1	0.857
PC-5	1	1	1	1	1	1	1	1.000
SCP-1	1	1	1	1	1	1	0	0.857
SCP-2	1	1	1	1	1	1	1	1.000
SCP-3	1	1	1	1	1	1	1	1.000
SCP-4	1	1	1	0	1	1	1	0.857
SR-1	1	1	1	1	1	1	1	1.000
SR-2	1	1	1	1	0	1	1	0.857
SR-3	1	1	1	1	1	1	1	1.000
SR-4	1	1	1	1	1	1	1	1.000
SS-1	1	1	1	1	1	1	1	1.000
SS-2	1	1	1	1	1	1	1	1.000
SS-3	1	1	1	1	1	1	1	1.000
SS-4	1	1	1	0	1	1	1	0.857
MA-1	0	1	1	1	1	1	1	0.857
MA-2	1	1	1	1	1	1	1	1.000
MA-3	1	1	1	1	1	1	0	0.857

*MR, Mindful Relaxation; PC, Physical Care; SCP, Self-Compassion and Purpose; SR, Supportive Relationships; SS, Supportive Structure; MA, Mindful Awareness*.

#### Exploratory Factor Analysis

**Figure 3:**
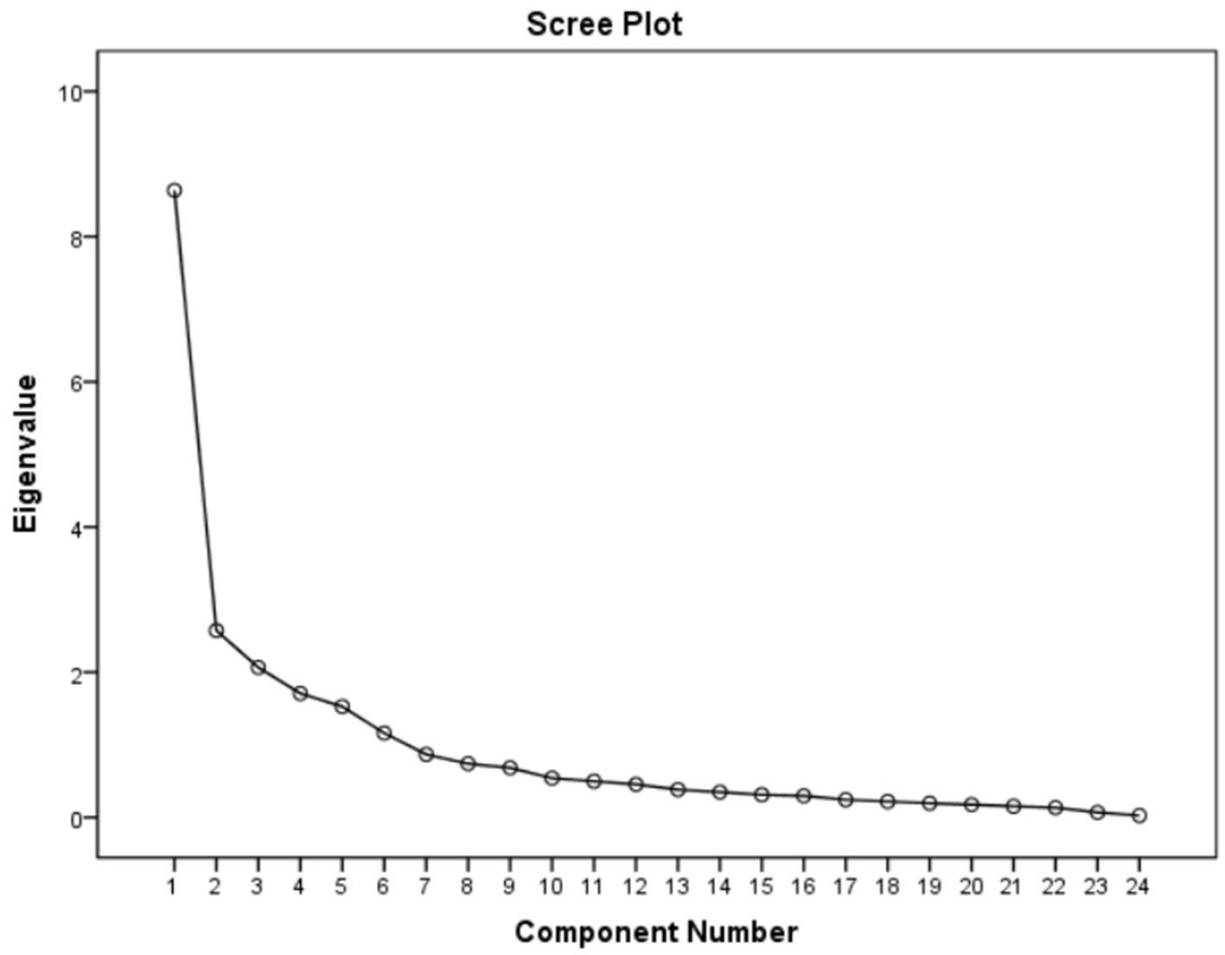
Screen plot of exploratory factor analysis for Chinese version of the B-MSCS.

The Kaiser-Meyer-Olkin Measure of Sampling Adequacy was 0.883, and the Bartlett test of sphericity was significant (χ^2^ = 9491.909; *P* < 0.001). Therefore, the matrix is not an identity matrix and is appropriate for factor extraction. According to the Kaiser's rule, six factors that explained a total of 73.623% of the variance had initial eigenvalues >1 each. The six-factor structure consistent with the original scale was further confirmed by the scree plot, as the descending tendency became weak after the sixth point (Figure 3). After the varimax rotation, the six factors explained 8.685, 9.787, 12.185, 13.422, 14.595, and 14.949% of the variance, respectively. Moreover, the factor loadings are also satisfactory and displayed in Table 5.

**Table 5:** Factor loadings of exploratory factor analysis for Chinese version of the B-MSCS.

**Item**	**Factor 1**	**Factor 2**	**Factor 3**	**Factor 4**	**Factor 5**	**Factor 6**
MR-1	-	-	0.816	-	-	-
MR-2	-	-	0.731	-	-	-
MR-3	-	-	0.834	-	-	-
MR-4	-	-	0.840	-	-	-
PC-1	0.779	-	-	-	-	-
PC-2	0.804	-	-	-	-	-
PC-3	0.733	-	-	-	-	-
PC-4	0.811	-	-	-	-	-
PC-5	0.851	-	-	-	-	-
SCP-1	-	-	-	0.875	-	-
SCP-2	-	-	-	0.780	-	-
SCP-3	-	-	-	0.885	-	-
SCP-4	-	-	-	0.608	-	-
SR-1	-	0.855	-	-	-	-
SR-2	-	0.874	-	-	-	-
SR-3	-	0.814	-	-	-	-
SR-4	-	0.851	-	-	-	-
SS-1	-	-	-	-	-	0.664
SS-2	-	-	-	-	-	0.579
SS-3	-	-	-	-	-	0.661
SS-4	-	-	-	-	-	0.648
MA-1	-	-	-	-	0.798	-
MA-2	-	-	-	-	0.866	-
MA-3	-	-	-	-	0.856	-

*MR, Mindful Relaxation; PC, Physical Care; SCP, Self-Compassion and Purpose; SR, Supportive Relationships; SS, Supportive Structure; MA, Mindful Awareness*.

#### Confirmatory Factor Analysis

**Figure 4:**
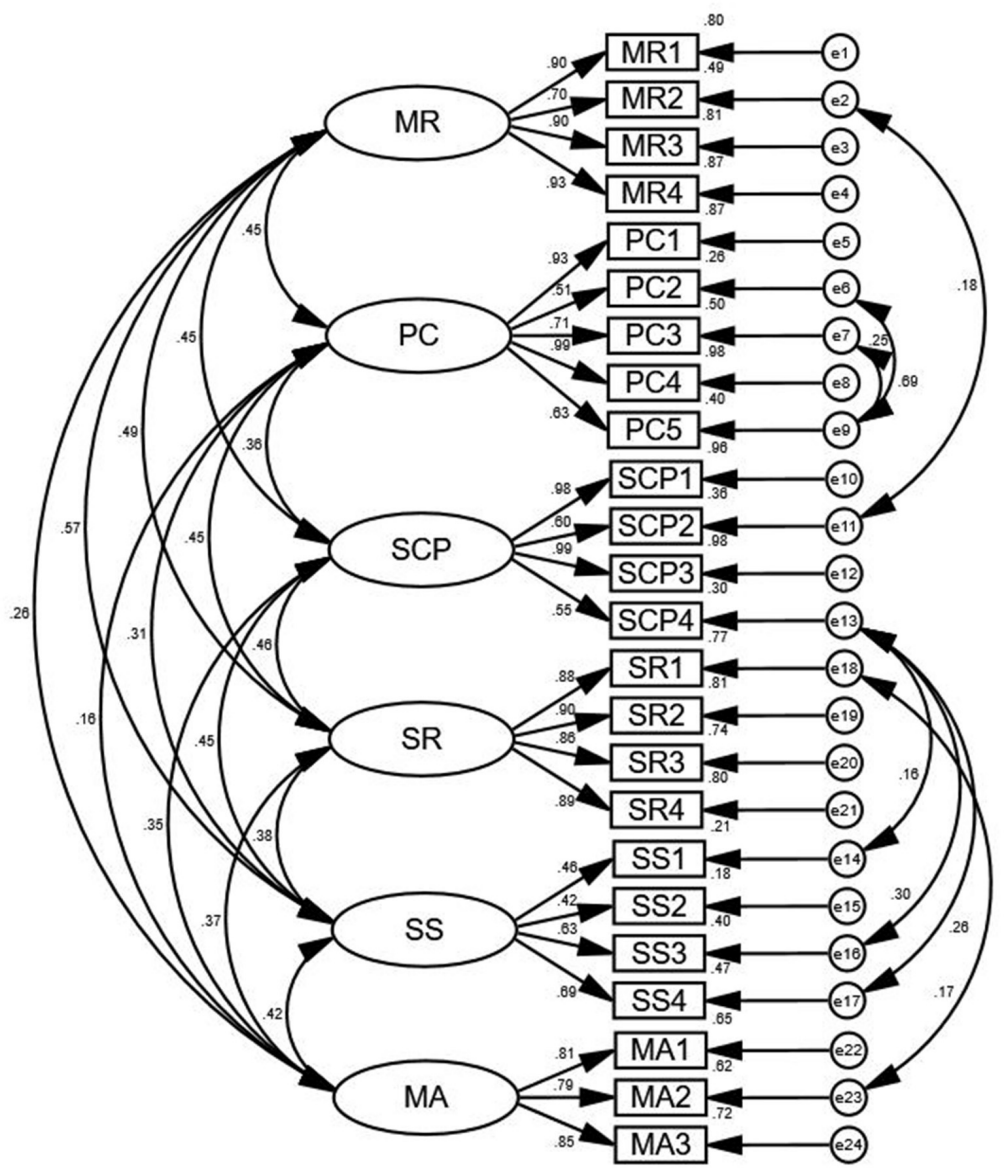
Standardized three-factor structural model of the BMSCS (*n* = 255). MR, Mindful Relaxation; PC, Physical Care; SCP, Self-Compassion and Purpose; SR, Supportive Relationships; SS, Supportive Structure; MA, Mindful Awareness.

The results of confirmatory factor analysis are shown in Figure 4. According to the modification indices (MI), the initial model was revised 7 times in order: e7 and e9, e6 and e9, e2 and e11, e13 and e14, e13 and e16, e13 and e17, e18 and e23, respectively. For the model fitness index, the chi-square/degree of freedom (χ^2^/df) was 2.431, the goodness-of-fit index (GFI) was 0.917, the adjusted goodness-of-fit index (AGFI) was 0.905, the root mean square error of approximation (RMSEA) was 0.043, the tucker lewis index (TLI) was 0.958, the comparative fit index (CFI) was 0.965, the incremental fit index (IFI) was 0.965, the parsimonious goodness-of-fit index (PGFI) was 0.703, and the parsimonious normed-of-fit index (PNFI) was 0.785.

## Discussion

### The Chinese Version of B-MSCS Has Suitable Distinction Among Hospice Nurses

In this study, based on the Brislin translation principle (Khalaila, 2013), nursing experts were invited to adjust the translation draft according to relevant guidelines and Chinese expression habits, and the Chinese version of B-MSCS was finally formed. The equivalence between the Chinese scale and the original scale was fully guaranteed. Through preliminary investigation, 40 hospice nurses believed that the semantic expression of the Chinese version of B-MSCS was clear, easy to understand, and the scale structure was reasonable. Furthermore, the CR of the items is much better than the standard value. The score of each item is moderately to highly correlated with the total score of the scale. Moreover, Cronbach's α value after deleting each item does not exceed the original value of the translated scale. All of the above indicated that 24 items of the Chinese version of B-MSCS can be retained and had better distinction.

### The Chinese Version of B-MSCS Has Suitable Reliability Among Hospice Nurses

Reliability analysis, a method of measuring the consistency and stability of the measured tool, is used to reflect the authenticity of the measured tool (Koo and Li, 2016). In this study, the reliability of the Chinese version of B-MSCS was evaluated from three aspects: internal consistency reliability, test-retest reliability and split-half reliability. The internal consistency expressed by Cronbach's α value reflects the homogeneity among all items in the scale (Anselmi et al., 2019). The results showed that the Cronbach's α value of the translated scale was 0.885, and the Cronbach's α value of each dimension was 0.770–0.854, which were slightly higher than the results of the original version (Cook-Cottone and Guyker, 2018). Test-retest reliability refers to the consistency of the results obtained by repeatedly measuring a group of subjects with a research tool. It reflects whether the measuring tool can stably measure the things or variables (Leppink and Pérez-Fuster, 2017). In this study, the test-retest reliability of the Chinese version of B-MSCS was better than the standard value, which shows that the scale is highly stable and could be reused among hospice nurses. In general, the Chinese version of B-MSCS has suitable reliability among hospice nurses.

### The Chinese Version of B-MSCS Has Suitable Validity Among Hospice Nurses

Validity refers to the degree to which the scale can reflect the expected research concepts (Kimberlin and Winterstein, 2008). In this study, the reliability of the Chinese version of B-MSCS was evaluated from content validity analysis and structure validity analysis. The Delphi method showed that I-CVI was 0.857–1.000 and S-CVI was 0.952, higher than 0.9 and 0.8 of the content validity reference value (Waltz et al., 2016). Furthermore, it is generally believed that the suitable structure validity is reflected in two aspects: (1) The factors extracted by exploratory factor analysis can explain 40.00% or more of the total data variation; (2) Each item has a higher load value on one common factor (>0.400) and a lower load value on other common factors. In this study, the six factors extracted by exploratory factor analysis can explain 73.623% of the total data variation. The factor attribution of all items is consistent with that of the original scale, and the factor loading of each item meets the above criteria (Cook-Cottone and Guyker, 2018). Meanwhile, the CFA results reported that the Chinese version of the B-MSCS had good fitting indexes, which were stronger than the fitting indexes reported for the original version (Cook-Cottone and Guyker, 2018). In general, the Chinese version of B-MSCS has suitable validity among hospice nurses.

## Limitation and Perspectives

There are some limitations to this study, which should be noted and discussed. Hospice nurses who are practicing or interested in mindfulness and self-care might be more likely to take the assessment. Social acceptability might bias the results of the study. Moreover, although the sample size was up to the standard in this study, the generalizability of these findings has some limits because of convenient sampling. Finally, although we have comprehensively verified the differentiation, reliability and validity of the Chinese version of the B-MSCS among hospice nurses, factors influencing mindful self-care have not been explored among hospice nurses. Therefore, it will be of primary importance for our next work.

## Conclusions

The English version of B-MSCS has been successfully introduced into China after translation and cultural adaptation, and its psychometric properties have also been verified among hospice nurses. Moreover, through factor analysis, it has been concluded that the Chinese version of B-MSCS has the suitable reliability and validity. Under the background lacking of hospice nurses and healthy China strategy, this provides an effective post-intervention measurement tool to improve the mindful self-care among Chinese hospice nurses and also provides a basis and precondition for the related research on the health belief level of hospice nurses.

## Data Availability Statement

The raw data supporting the conclusions of this article will be made available by the authors, without undue reservation.

## Ethics Statement

The studies involving human participants were reviewed and approved by The Ethics Committee of Jinzhou Medical University. The patients/participants provided their written informed consent to participate in this study.

## Author Contributions

All authors contributed to the design of the study and data collection. ZY drafted the manuscript after data collection and analysis, and HZ revised it critically for important intellectual content. All the other co-authors also made significant contributions to the revision of the manuscript.

## Conflict of Interest

The authors declare that the research was conducted in the absence of any commercial or financial relationships that could be construed as a potential conflict of interest.

## Publisher's Note

All claims expressed in this article are solely those of the authors and do not necessarily represent those of their affiliated organizations, or those of the publisher, the editors and the reviewers. Any product that may be evaluated in this article, or claim that may be made by its manufacturer, is not guaranteed or endorsed by the publisher.
